# Alcohol exposure before and during pregnancy is associated with reduced fetal growth: the Safe Passage Study

**DOI:** 10.1186/s12916-023-03020-4

**Published:** 2023-08-23

**Authors:** Marin Pielage, Hanan El Marroun, Hein J. Odendaal, Sten P. Willemsen, Manon H. J. Hillegers, Eric A. P. Steegers, Melek Rousian

**Affiliations:** 1 Department of Obstetrics and Gynecology, Erasmus MC, University Medical Center, Room Sp-4469, PO Box 2040, 3000 CA Rotterdam, The Netherlands; 2 Department of Child and Adolescent Psychiatry/Psychology, Erasmus MC, University Medical Center, Sophia Children’s Hospital, 3000 CB Rotterdam, the Netherlands; 3 Department of Psychology, Education and Child Studies – Erasmus School of Social and Behavioral Sciences, Erasmus University Rotterdam, Rotterdam, The Netherlands; 4 Department of Obstetrics and Gynaecology, Faculty of Medicine and Health Sciences, Stellenbosch University, Stellenbosch, South Africa; 5 Department of Biostatistics, Erasmus MC, University Medical Center, PO Box 2040, 3000 CA Rotterdam, The Netherlands

**Keywords:** Alcohol, Fetal alcohol spectrum disorders, Fetal growth restriction, Pregnancy, Prenatal alcohol exposure, Prenatal ultrasonography

## Abstract

**Background:**

Prenatal alcohol exposure (PAE) is a worldwide public health concern. While PAE is known to be associated with low birth weight, little is known about timing and quantity of PAE on fetal growth. This study investigated the association between periconceptional and prenatal alcohol exposure and longitudinal fetal growth, focusing on timing and quantity in a high exposure cohort.

**Methods:**

The Safe Passage Study was a prospective cohort study, including 1698 pregnant women. Two-dimensional transabdominal ultrasound examinations were performed to measure fetal femur length, abdominal and head circumference, and biparietal diameter, at three time points during pregnancy. Estimated fetal weight and *Z*-scores of all parameters were calculated. Trimester-specific alcohol exposure was assessed using the Timeline Followback method. To investigate the associations of specific timing of PAE and fetal growth, two models were built. One with alcohol exposure as accumulative parameter over the course of pregnancy and one trimester specific model, in which PAE was separately analyzed. Linear mixed models adjusted for potential confounders were applied with repeated assessments of both alcohol exposure and fetal growth outcomes.

**Results:**

This study demonstrated that periconceptional and prenatal alcohol exposure were associated with reduced fetal growth. Effect sizes are displayed as estimated differences (*ED)* in *Z*-score and corresponding 95% confidence intervals (95% CIs). When investigated as accumulative parameter, PAE was related to a smaller femur length (*ED*_*30*_; − 0.13 (95% CI; − 0.22; − 0.04), *ED*_*36*_; − 0.14 (95% CI; − 0.25; − 0.04)) and a smaller abdominal circumference (*ED*_*36*_; − 0.09 (95% CI; − 0.18; − 0.01)). Periconceptional alcohol exposure was associated with a smaller abdominal circumference (*ED*_*30*_; − 0.14 (95% CI; − 0.25; − 0.02), *ED*_*36*_; − 0.22 (95% CI; − 0.37; − 0.06)) and a smaller estimated fetal weight (*ED*_*36*_; − 0.22 (95% CI; − 0.38; − 0.05)). Second trimester alcohol exposure was associated with a smaller abdominal circumference (*ED*_*30*_; − 0.49 (95% CI; − 0.86; − 0.12), *ED*_*36*_; − 0.70 (95% CI; − 1.22; − 0.17)) and estimated fetal weight (*ED*_*30*_; − 0.54 (95% CI; − 0.94; − 0.14), *ED*_*36*_; − 0.69 (95% CI; − 1.25; − 0.14)). No additional association of binge drinking was found besides the already observed association of PAE and fetal growth.

**Conclusions:**

This study demonstrated that PAE negatively affects fetal growth, in particular when exposed during the periconception period or in second trimester. Our results indicate that potential negative consequences of PAE are detectable already before birth. Therefore, healthcare providers should actively address and discourage alcohol use during pregnancy.

**Supplementary Information:**

The online version contains supplementary material available at 10.1186/s12916-023-03020-4.

## Background

Prenatal alcohol exposure (PAE) is a public health concern, and despite worldwide efforts to avoid PAE, the estimated global prevalence of alcohol consumption during pregnancy is still 10%. The prevalence of alcohol consumption during pregnancy varies between countries, being on average the lowest (0.2%) in countries in the Eastern-Mediterranean region, and on average the highest in countries in the European region (25%) [1]. In general, the South African population, including men and women, has one of the highest levels of alcohol consumption (28%), including heavy drinking [2, 3]. The Western Cape is known to be the most problematic area, with the prevalence of any alcohol consumption during pregnancy reaching 38% [4]. PAE has been linked to poor pregnancy outcomes: miscarriage, stillbirth, and premature birth [5–8]. Furthermore, substantial maternal alcohol consumption causes fetal alcohol spectrum disorders (FASD), a continuum of neurodevelopmental disabilities, craniofacial and somatic anomalies, with a global prevalence of 7.7 per 1000 and 111.1 per 1000 in specific South African communities [1, 9, 10]. Maternal alcohol consumption leads to fetal exposure by placental diffusion and distribution in the fetal compartment by amniotic fluid accumulation. Additionally, low fetal metabolic enzyme concentrations delays alcohol elimination and along with amniotic reuptake, results in prolonged exposure and potential adverse effects [11, 12].

Although PAE is thoroughly studied, information on specific associations of timing and quantity of PAE with fetal growth is limited. Many studies on growth impairment due to PAE focus on birth weight and have inconclusive results [8, 13–15]. Since birth weight is a single measurement, and provides little information on intrauterine growth, it is insufficient to interpret fetal growth. Few studies investigated the association between PAE and fetal growth using longitudinal data, reporting no differences between alcohol-exposed fetuses and controls [16–18]. However, these studies were performed in small samples, included low exposure groups, or did not investigate associations between periconceptional alcohol exposure and fetal growth later in pregnancy. Periconceptional alcohol exposure was shown to be associated with reduced embryonic growth, reflected by a smaller crown-rump length at 6 and 12 weeks of gestation [19]. Most studies use alcohol exposure as categorized variable, providing limited information on timing and quantity of PAE [16–19]. A continuous measure provides detailed information on prenatal time-windows in which alcohol could influence fetal growth. Finally, few human studies examined binge drinking (i.e., drinking ≥ 4 consumptions per occasion) during pregnancy, which may cause a higher peak blood concentration (PBC) in a short time span than regular drinking [20, 21]. Animal studies have shown that PBC may be more important than the total daily alcohol dose influencing fetal development [22]. As such, we hypothesized that binge drinking during pregnancy has an additional negative effect on fetal growth than moderate alcohol exposure only. From this background, it is important to use detailed and longitudinal information about both alcohol exposure and fetal growth. Therefore, in the current study, we used data from a unique study population from Cape Town, South Africa, which consisted of a large sample size, with a high percentage of pregnant women drinking alcohol in all three trimesters (longitudinally) and with heterogeneity in alcohol consumption levels (i.e., low, moderate, high and binge drinking) [23]. We aimed to investigate the association of both periconceptional and prenatal alcohol exposure in relation to longitudinal fetal growth and pregnancy outcomes, focusing on timing and quantity (including binge drinking) of alcohol exposure. We hypothesized that periconceptional and prenatal alcohol exposure is negatively associated with both fetal growth trajectories and pregnancy outcomes.

## Methods

### Study design

This study was embedded in the Safe Passage Study, a prospective cohort study, conducted between 2007 and 2016 [23]. The total cohort included 12,000 pregnant women, recruited from predefined communities at high risk for prenatal alcohol use (USA and South Africa). The detailed study protocol is described elsewhere [23]. Previous studies in the South African arm of the cohort showed a reduction in birth weight *Z*-scores in neonates prenatally exposed to alcohol [24, 25]. However, PAE as variable was categorized, causing difficulties in interpretation of PAE-effects. The current study, restricted to the South African arm of the study, investigated PAE as continuous variable in a longitudinal manner, focusing on timing and quantity, clarifying relationships more efficiently.

In a randomly selected subset (*n* = 1928) of the Safe Passage cohort recruited in South Africa, additional measures were collected. Women in this so-called *embedded protocol* were enrolled before 24 weeks of gestation [23]. After enrollment, three remaining visits occurred at 20–24, 28–32, and after 34 weeks of gestation. All women gave informed consent.

### Study population

Within the sub-cohort (*n* = 1928), we excluded women with missing ultrasound or growth data (*n* = 91) and second or third participations in the study (*n* = 81). Pregnancies with congenital anomalies or different growth patterns due to other reasons than alcohol exposure were additionally excluded: twin pregnancies (*n* = 12, 24 fetuses), miscarriages (*n* = 3), congenital anomalies (*n* = 4), stillbirths (*n* = 25), and terminations of pregnancy (*n* = 3) (Additional file 1: Figure S1). The final study population included 1698 women with singleton pregnancies.

### Data collection

#### Alcohol and tobacco exposure

Information on maternal alcohol consumption was collected through interviews performed by trained research staff, using an adapted version of the Timeline Followback method (TLFB) [26]. This method was contemplated as one of the most strict methods in self-reported exposure assessment, using calendar worksheets and visual prompts to support participants in recall about their alcohol consumption [27]. Maternal alcohol consumption periods were defined as follows: within the periconception period, defined as 15 days prior to and 15 days after the last menstrual period (LMP), first trimester was defined as gestational days 0 until 97 (equal to 13 + 6 weeks of gestation), second trimester as gestational days 98 until 195 (equal to 14 + 0 until 27 + 6 weeks of gestation), and third trimester as ranging from gestational day 196 until delivery (equal to 28 + 0 weeks of gestation until delivery). It should be noted there is a slight overlap between the periconception period and first trimester. Maternal alcohol consumption during the periconception period was collected at the enrollment visit. For each trimester, collected during the following prenatal visits, alcohol consumption data were collected based on the last reported drinking day and 30 days prior. For each drinking day, detailed information on the type of drink, the number and size of drinks, drinks including ice, and the duration of drinking was collected. The total amount of alcohol in grams was converted into standard drinks during the periconception period and per trimester separately [28]. Furthermore, the average amount of alcohol-use in grams per day during pregnancy as a whole and for the periconception period and each trimester were calculated. By definition, one standard drink contains 14 g of alcohol, and binge drinking is defined as drinking ≥ 4 alcoholic drinks per occasion [29, 30]. Maternal binge drinking was calculated as the total amount of binge-moments during pregnancy. The prevalence of total PAE in this study was 62%, and 27% of women reported binge drinking. Fetuses not exposed to maternal alcohol consumption were referred to as controls.

Since alcohol-using women were more likely to smoke cigarettes, which is known to reduce fetal growth, we also investigated maternal tobacco use during pregnancy [31, 32]. This was investigated using questionnaires with graduated frequency response options (e.g., none, monthly or less, 2–4 days/month, 2–3 days/week, 4–6 days/week, and 7 days/week) and the number of cigarettes smoked per day, covering a 30-day reference period prior to the last smoking day. The average amount of cigarettes per day was calculated.

#### Fetal growth, pregnancy, and pregnancy outcomes

Trained sonographers performed two-dimensional ultrasound examinations trans abdominally using a Voluson E8 ultrasound machine (GE Healthcare) with a RAB 4–8 3D transducer, applying internationally standardized protocols. Pregnancy dating occurred at the first prenatal visit. Between 6 + 0 and 13 + 6 weeks of gestation, the fetal crown-rump-length (CRL) was used. From 14 + 0 weeks onwards, fetal head circumference (HC), fetal biparietal diameter (BPD), fetal abdominal circumference (AC), and fetal femur length (FL) were used for pregnancy dating, as well as for fetal growth measurements [33]. Estimated fetal weight (EFW) was calculated based on HC, BPD, AC, and FL. All fetal growth outcome measures (including HC, BPD, AC, FL, EFW, and birth weight) were transformed into *Z*-scores using the INTERGROWTH-21^st^ project standard formulas, correcting for exact gestational age (GA) in weeks [34, 35]. Fetal growth *Z*-scores were treated as continuous longitudinal variables.

Information on maternal hypertensive disorders (systolic blood pressure > 140 mmHg or diastolic blood pressure > 90 mmHg), birth weight, and preterm birth (< 37 weeks of gestation) were collected from medical records.

#### Covariates

Potential covariates were selected a priori based on previous studies [16, 36, 37]. Self-reported maternal characteristics (at enrollment) included age, parity, obstetric and medical history, years of education, and monthly income in South African Rand [23]. Also, maternal anxiety was collected at enrollment, using the state-anxiety subscale from the Spielberger state trait anxiety scale [38, 39]. Maternal depression, collected at the enrollment visit, was scored using the Edinburgh postnatal depression scale (EPDS) [40]. Maternal mid-upper arm circumference (MUAC) and self-reported other substance use (e.g., marijuana and methamphetamine) were collected each visit, of which procedures were described previously [23, 41].

### Statistical analysis

To evaluate non-response and investigate representativeness of the study sample for the entire study population, baseline characteristics between included and excluded women were compared (Additional file 2: Table S1). Baseline characteristics of the alcohol exposed group were compared to controls (Table 1). Continuous variables were analyzed using Student’s *t*-test (normal distributed) or Kruskal–Wallis test (non-normal distributed), categorical variables with *χ*^2^ tests.

**Table 1:** Baseline characteristics of the study population

	All study participants	No alcohol exposure (*n* = 594)	Alcohol exposure (*n* = 1104)	
*Maternal characteristics*				*p-*value
Age (years)	**24.8 ± 0.14**	**25.3 ± 0.25**	**24.5 ± 0.17**	**.04**
Nullipara	724 (42.6%)	238 (40.1%)	486 (44.0%)	.34
BMI (kg/m^2^)	**25.3 ± 0.15**	**25.8 ± 0.26**	**24.9 ± 0.17**	** < .01**
MUAC (mm)	**276.9 ± 1.16**	**281.9 ± 2.08**	**274.0 ± 1.36**	** < .01**
*Exposure to substances*^a^				
Mean alcohol intake periconception period (g/day)	2.3 ± 0.16	-	3.61 ± 0.25	-
Mean alcohol intake 1st trimester (g/day)	0.91 ± 0.1	-	1.40 ± 0.10	-
Mean alcohol intake 2nd trimester (g/day)	0.58 ± 0.04	-	0.89 ± 0.07	-
Mean alcohol intake 3rd trimester (g/day)	0.27 ± 0.02	-	0.41 ± 0.04	-
Tobacco use during pregnancy (% users)	**1120 (66.0%)**	**311 (52.4%)**	**809 (73.2%)**	** < .01**
Other drug use^b^	**195 (11.5%)**	**33 (5.6%)**	**162 (14.7%)**	** < .01**
*Socioeconomic characteristics*				
Ethnicity^c^				
Black	4 (0.2%)	2 (0.3%)	2 (0.2%)	-
Cape colored (mixed ancestry)	1694 (99.8%)	592 (99.7%)	1102 (99.8%)	-
Income (monthly, South African Rand)	911.7 ± 16.7	967.0 ± 26.9	906.6 ± 19.9	.13
Employment (% employed)	558 (32.9%)	199 (33.5%)	359 (32.5%)	.13
Education (years)	10.1 ± 0.04	10.18 ± 0.07	10.09 ± 0.05	.28
*Prenatal psychopathology*				
Anxiety scores	30.8 ± 0.2	**30.1 ± 0.4**	**31.3 ± 0.31**	**.02**
Depression (% above cut-off)	**854 (50.3%)**	**274 (46.1%)**	**580 (52.5%)**	** < .01**
*Pregnancy outcomes*				
Gestational diabetes (% disease)	13 (0.8%)	7 (1.2%)	6 (0.5%)	.15
Hypertensive disorder in pregnancy (%)	197 (11.6%)	70 (11.8%)	127 (11.5%)	.70
Birth weight (g)^d^	**3012 ± 13.8**	**3057 ± 23.5**	**2988 ± 16.9**	**.01**
Fetal sex (% female)	872 (51.4%)	295 (49.7%)	577 (52.3%)	.46
Preterm birth (% preterm)	204 (12%)	74 (12.5%)	130 (11.8%)	.70
GA at birth (in weeks)	38.9 ± 0.05	39.0 ± 0.09	38.9 ± 0.06	.29

Continuous data are presented as means ± standard errors; categorical variables are presented in numbers (%). Comparisons between non-exposed and exposed were performed using Student’s *t*-test for normal distributed continuous variables, Mann–Whitney *U* test for non-normal distributed continuous variables, and Pearson *χ*^2^ tests for categorical variables

*Abbreviations: BMI*, body mass index; *GA*, gestational age; *MUAC*, mid- upper arm circumference

^a^Mean alcohol intake was not statistically tested, since the reference group was non-exposed

^b^Other drugs = marijuana or methamphetamine

^c^Ethnicity was not compared due to very small subgroups

^d^Birth weight was missing in the non-exposed group (*n* = 5) and the alcohol-exposed group (*n* = 10)

Very few variables were missing, except monthly income (26% missing), for which multiple imputation by chained equations was used (25 datasets, Additional file 2: Table S2) [42].

To investigate the association between PAE and fetal growth or birth weight *Z*-scores, linear mixed models were applied. We first investigated the association of PAE as accumulative parameter over the course of pregnancy and fetal growth *Z*-scores, using a model in which average alcohol consumption (grams/day) during pregnancy and GA were added as predictors (model 1, accumulation model). Second, we built a model in which PAE per trimester was separated, to investigate whether exposure in any specific trimester was crucial in fetal growth. In this model, four different exposure periods (periconception period, trimester 1 (T1), trimester 2 (T2), and trimester 3 (T3)) and GA were added as predictors (model 2, trimester-specific model). Since third trimester alcohol exposure cannot influence second trimester growth, we modeled that third trimester PAE could only influence third trimester growth measurements. First and second trimester PAE can influence both second and third trimester growth measurements. Similar methods were applied to investigate associations between PAE and birth weight *Z*-scores. Our secondary analysis examined the association of binge drinking with fetal growth, applied as bingers compared to non-drinkers and to drinkers, but non-bingers (model 3, binge drinking model). Predictors in this model were PAE as accumulative parameter and total binge drinking moments during pregnancy. To explore the association of smoking with fetal growth, a fourth model was built adding smoking as single predictor, as well as simultaneous tobacco and alcohol exposure (model 4, tobacco co-use model). In all analyses, growth trajectories of alcohol exposed fetuses were compared to those of controls using linear mixed models, by calculating estimated *Z*-score differences per growth measurement. Lastly, the associations between PAE and hypertensive disorders or preterm birth, both dichotomous outcomes, were investigated using logistic regression analysis.

All models, except for model 3, were adjusted for fetal sex, maternal age, MUAC, parity, years of education, monthly income, other drug use (marijuana and/or methamphetamine), anxiety, and smoking (in models 1 and 2). Model 3 was only adjusted for fetal sex. For interpretability purposes, we estimated *Z*-score differences *(ED)* with 95% confidence intervals (CIs) using preset situations, all calculated for 20, 30, and 36 weeks of gestation, except for the binge drinking model, in which we calculated beta’s (*β*) and 95% CIs. Model 3 was built to investigate if binge drinking has an additional association with fetal growth. For interpretability purposes, we calculated uncorrected *β*s, indicating the additional increase or decrease in fetal growth per unit increase in alcohol consumption.

In these preset situations, variables were included as follows: alcohol exposure = 1 standard drink/day, mean tobacco exposure = 10 cigarettes/day (only in model 4), fetal sex = “male”, maternal age = 25 years, MUAC = 277 mm, parity = 1, education = 10 years, monthly income = 910 South African Rand, other drug use = “yes”, anxiety score = 31, average cigarette exposure/day (to adjust for smoking in models 1 and 2) = 3.17.

In all analyses, the SPSS statistical software version 25.0 and R statistical software version 4.1.0 for Windows were used. A *p*-value ≤ 0.05 was considered statistically significant.

## Results

### Descriptive statistics

Baseline characteristics of the study population are shown in Table 1. Of all women consuming alcohol during pregnancy, most women consume alcohol during the periconception period (46%) or first trimester (69%). The distribution of alcohol consumption during each period is displayed in Additional file 3: Figure S2. After first trimester, alcohol consumption decreases to 54% in second trimester and 31% during third trimester. Alcohol-consuming pregnant women were younger than controls (24.5 ± 0.17 vs. 25.3 ± 0.25, *p* < 0.05) and had a lower BMI (24.9 ± 0.17 vs. 25.8 ± 0.26, *p* < 0.01) and a smaller MUAC (274.0 ± 1.36 vs. 281.9 ± 2.08, *p* < 0.01). Moreover, they were more likely to smoke cigarettes (73.2% vs. 52.4%, *p* < 0.01) and use other drugs (e.g., marijuana or methamphetamine, 14.7% vs. 5.6%, *p* < 0.01). Lastly, alcohol-using pregnant women scored on average higher on anxiety symptoms (31.3 ± 0.31 vs. 30.1 ± 0.4, *p* < 0.05) and more often had depressive symptoms, when compared to controls (52.5% vs. 46.1%, *p* < 0.05).

### Accumulative alcohol exposure and fetal growth

The results of the accumulation model are shown in Tables 2 and  3. In this model, PAE was associated with a smaller a FL at 30 and 36 weeks (*ED*_*30*_; − 0.13 (95% CI; − 0.22; − 0.04), *ED*_*36*_; − 0.14 (95% CI; − 0.25; − 0.04)). Moreover, PAE was also associated with a smaller AC at 36 weeks of gestation (*ED*_*36*_; − 0.09 (95% CI; − 0.18; − 0.01), Table 2). Accumulative PAE was associated with a smaller birth weight *Z*-score (*ED*; − 0.18 (95% CI; − 0.29; − 0.07) when compared to controls (Table 3).

**Table 2:** Associations between overall alcohol exposure and fetal growth (model 1, accumulation model)

		Unadjusted model			Adjusted model^a^	
	*n*	*ED* Z-score (95% CI)	*p-*value	*n*	*ED* Z-score (95% CI)	*p*-value
**BPD**						
*20 weeks*	1151	.05 (− .07; .17)	.42	1142	.05 (− .07; .17)	.41
*30 weeks*		− .06 (− .16; .03)	.18		− .05 (− .14; .04)	.29
*36 weeks*		** − .13 (− .25; − .02)**	**.02**		− .11 (− .22; .00)	.05
**HC**						
*20 weeks*	1106	− .02 (− .13; .10)	.77	1097	− .01 (− .12; .11)	.92
*30 weeks*		− .07 (− .16; .02)	.11		− .06 (− .15; .02)	.16
*36 weeks*		− .10 (− .21; − .01)	.07		− .09 (− .20; .01)	.09
**AC**						
*20 weeks*	948	.00 (− .10; .10)	.99	940	− .003 (− .10; .10)	.96
*30 weeks*		− .07 (− .14; .01)	.08		− .05(− .13; .02)	.16
*36 weeks*		** − .10 (− .20; − 0.01)**	**.03**		** − .09 (− .18; − .01)**	**.01**
**FL**						
*20 weeks*	1396	− .11 (− .22; .01)	.07	1387	− .11 (− .22; .01)	.07
*30 weeks*		** − .15 (− .24; − .06)**	** < .01**		** − .13 (− .22; − .04)**	** < .01**
*36 weeks*		** − .17 (− .28; − .07)**	** < .01**		** − .14 (− .25; − .04)**	** < .01**
**EFW**						
*20 weeks*	816	− .02 (− .13; .09)	.72	839	− .01 (− .12; .10)	.88
*30 weeks*		− .07 (− .16; .01)	.08		− .06 (− .14; .02)	.18
*36 weeks*		− .10 (− .21; − .00)	.05		− .08 (− .18; .02)	.11

Estimated differences *(ED)* in *Z*-score were calculated for preset situations at GA 20, 30 and 36 weeks comparing an exposed fetus to a non-exposed fetus

Situation outlined:

Exposure = on average 1 standard drink (14 g of alcohol) per day

*n* is displayed per outcome, based on availability of alcohol exposure, growth measures, and gestational age

*Abbreviations: AC*, (fetal) abdominal circumference; *BPD*, (fetal) biparietal diameter; *CI*, confidence interval; *EFW*, estimated fetal weight; *FL*, (fetal) femur length; *HC*, (fetal) head circumference

^a^Adjusted model settings for comparison; maternal age = 25 years, monthly income = 910 SA Rand, anxiety score = 31, parity = 1, education = 10 years, fetal sex = "male", other drug use = "yes", average cigarette exposure per day = 3.17, MUAC = 277 mm

**Table 3:** Associations between overall and trimester specific alcohol exposure and birth weight

	**BW—unadjusted**		**BW—adjusted**^**a**^	
	*ED* Z-score (95% CI)	*p-*value	*ED* Z-score (95% CI)	*p-*value
**PAE accumulative**	** − .21 (− .32; − .09)**	** < .01**	** − .18 (− .29; − .07)**	** < .01**
**Periconceptional**	− .16 (− .34; .03)	.11	− .10 (− .28; .09)	.30
**T1**	.11 (− .45; .67)	.69	− .09 (− .63; .45)	.75
**T2**	** − .76 (− 1.41; − .10)**	**.02**	** − .76 (− 1.41; − .12)**	**.02**
**T3**	1.18 (− .10; 2.46)	.07	**1.30 (.06; 2.54)**	**.04**

Estimated differences *(ED)* in Z-score were calculated for preset situations at birth at 40 weeks of gestation, comparing and exposed and non-exposed fetus

Situation outlined: exposure to on average 1 standard drink (14 g of alcohol) per day

*Abbreviations: BW*, birth weight; *CI*, confidence interval; *ED*, estimated difference; *PAE*, prenatal alcohol exposure; T1, trimester 1; *T2*, trimester 2; *T3*, trimester 3

^a^Adjusted model settings for comparison; maternal age = 25 years, income = 910 SA Rand, anxiety score = 31, parity = 1, education = 10 years, fetal sex = “male”, other drug use = “yes”, MUAC = 277 mm

### Trimester-specific alcohol exposure and fetal growth

In the trimester-specific model (model 2, Table 4), periconceptional alcohol exposure was associated with a smaller AC at 30 and 36 weeks (*ED*_*30*_; − 0.14 (95%CI; − 0.25; − 0.02), *ED*_*36*_; − 0.22 (95% CI; − 0.37; − 0.06)).

**Table 4:** Associations between trimester specific alcohol exposure and fetal growth (model 2, trimester-specific model)

	*n*	**Periconception period**		**T1**		**T2**		**T3**	
		*ED* Z-score (95% CI)	*p-*value	*ED* Z-score (95% CI)	*p-*value	*ED* Z-score (95% CI)	*p-*value	*ED* Z-score (95% CI)	*p-*value
**BPD**									
*20 weeks*	1097	.15 (− .02; .33)	.08	− .07 (− .55; .41)	.78	− .30 (− .83; .23)	.27	−	−
*30 weeks*		.05 (− .08; .19)	.44	− .09 (− .48; .29)	.64	− .41 (− .82; .01)	.06	− .01 (− .27; .24)	.91
*36 weeks*		− .00 (− .18; .17)	.96	− .11 (− .59; .37)	.66	− .47 (− 1.02; .08)	.10	− .06 (− 1.06; .95)	.91
**HC**									
*20 weeks*	1056	.14 (− .02; .31)	.09	− .27 (− .72; .19)	.25	− .10 (− .60; .39)	.69	−	−
*30 weeks*		.04 (− .09; .17)	.54	− .10 (− .46; .25)	.57	− .29 (− .67; .10)	.14	.05 (− .20; .30)	.68
*36 weeks*		− .02 (− .19; .15)	.80	− .01 (− .46; .45)	.98	− .40 (− .92; − .12)	.13	.21 (− .79; 1.21)	.68
**AC**									
*20 weeks*	900	− .01 (− .15; .14)	.95	− .14 (− .54; .26)	.50	− .15 (− .57; .27)	.49	−	−
*30 weeks*		** − .14 (− .25; − .02)**	**.02**	.17 (− .16; .49)	.31	** − .49 (− .86; − .12)**	** < .01**	.20 (− .01; .40)	.06
*36 weeks*		** − .22 (− .37; − .06)**	** < .01**	.35 (− .08; .77)	.11	** − .70 (− 1.22; − .17)**	** < .01**	.78 (− .04; 1.60)	.06
**FL**									
*20 weeks*	1336	− .07 (− .24; .10)	.41	− .14 (− .60; .32)	.55	− .03 (− .52; .47)	.92	-	** − **
*30 weeks*		− .10 (− .24; .03)	.14	.06 (− .30; .44)	.73	− .23 (− .62; .17)	.27	** − .20 (− .39; − .01)**	**.04**
*36 weeks*		− .12 (− .28; .04)	.14	.19 (− .25; .62)	.39	− .34 (− .84; .15)	.18	** − .81 (− 1.57; − .04)**	**.04**
**EFW**									
*20 weeks*	807	.04 (− .11; .19)	.60	− .16 (− .58; .26)	.46	− .28 (− .73; .18)	.23	−	−
*30 weeks*		− .12 (− .24; .00)	.06	.16 (− .19; .51)	.37	** − .54 (− .94; − .14)**	** < .01**	.18 (− .05; .41)	.13
*36 weeks*		** − .22 (− .38; − .05)**	**.01**	.35 (− .10; .80)	.12	** − .69 (− 1.25; − .14)**	**.02**	.71 (− .21; 1.64)	.13

Estimated differences *(ED)* in *Z*-score were calculated for preset situations at GA 20, 30, and 36 weeks, comparing an exposed fetus to a non-exposed fetus. Results of the adjusted model are displayed

Situation outlined:

Exposure = on average 1 standard drink (14 g of alcohol) per day

Adjusted model settings for comparison; maternal age = 25 years, monthly income = 910 SA Rand, anxiety score = 31, parity = 1, education = 10 years, fetal sex = "male", other drug use = "yes", average cigarette exposure per day = 3.17, MUAC = 277 mm

*n* is displayed per outcome, based on availability of growth measures, alcohol exposure, and gestational age

*Abbreviations: AC*, (fetal) abdominal circumference; *BPD*, (fetal) biparietal diameter; *CI*, confidence interval; *EFW*, estimated fetal weight; *FL*, (fetal) femur length; *HC*, (fetal) head circumference

Exposure during the periconception period was also associated with a smaller EFW at 36 weeks (*ED*_*36*_; − 0.22 (95% CI; − 0.38; − 0.05)) but was not associated with BPD, HC, FL, and birth weight.

In this study, first trimester PAE was not associated with fetal growth or birth weight (Tables 3 and 4). Second trimester alcohol exposure was associated with a smaller AC (*ED*_*30*_; − 0.49 (95% CI; − 0.86; − 0.12), *ED*_*36*_; − 0.70 (95% CI; − 1.22; − 0.17)) and smaller EFW (*ED*_*30*_; − 0.54 (95% CI; − 0.94; − 0.14), *ED*_*36*_; − 0.69 (95% CI; − 1.25; − 0.14)) at 30 and 36 weeks of gestation. As expected, BPD, HC, and FL were smaller in second trimester exposed fetuses, although non-significant.

Besides, second trimester alcohol exposure was associated with lower birth weight (*ED*; − 0.76 (95% CI; − 1.41; − 0.12)). For illustrative purposes, we included a figure in supplementary material (Additional file 4: Fig S3), to clarify all mentioned differences between alcohol exposed fetuses and controls. Third trimester alcohol exposure was associated with a smaller FL (*ED*_*30*_; − 0.13 (95% CI; − 0.22; − 0.04), *ED*_*36*_; − 0.14 (95% CI; − 0.25; − 0.04)) at 30 and 36 weeks and with increased birth weight (*ED*; 1.30 (95% CI; 0.06; 2.54), Table 3).

### Binge drinking, tobacco smoking and fetal growth

As shown in Table 5, binge drinking was not associated with fetal growth. Table 6 depicts the tobacco co-use model. Smoking alone was not associated with fetal growth, indicated by very small *Z*-score differences, whereas simultaneous tobacco smoking and PAE was related to a smaller FL at 30 and 36 weeks of gestation *ED*_*30*_; − 0.13 (− 0.22; − 0.04), *ED*_*36*_; − 0.14 (− 0.25; − 0.04)) (Table 6). All other growth measures, including BPD, HC, AC, and EFW, were not associated.

**Table 5:** Associations between binge drinking and fetal growth (model 3, binge drinking model)

	Binge vs. non drinkers^a^		Binge vs. non-binge drinkers ^b^			Binge drinking total study population	
	*β (95% CI)*	*p-*value	*β (95% CI)*	*p-*value		*β (95% CI)*	*p-*value
**BPD—Z**	− .01 (− .04; .02)	.52	− .02 (− .04; .01)		.25	− .02 (− .04; .01)	.23
**HC—Z**	− .01 (− .04; .02)	.47	− .02 (− .04; .01)		.23	− .02 (− .05; .01)	.21
**AC—Z**	− .01 (− .04; .01)	.22	− .01 (− .04; .01)		.26	− .01 (− .04; .01)	.25
**FL—Z**	− .002 (− .03; .03)	.68	− .001 (− .03; .03)		.94	− .01 (− .03; .03)	.95
**EFW—Z**	− .02 (− .04; .01)	.20	− .02 (− .04; .01)		.19	− .02 (− .04; .01)	.18

All analyses were adjusted for fetal sex

*Abbreviations: AC*, (fetal) abdominal circumference; *BPD*, (fetal) biparietal diameter; *CI*, confidence interval; *EFW*, estimated fetal weight; *FL*, (fetal) femur length; *HC*, (fetal) head circumference

^a^Fetuses exposed to binge drinking are already exposed to alcohol in pregnancy. Binge-exposed fetuses were compared to non-exposed fetuses

^b^Binge-exposed fetuses were compared to alcohol-exposed (but not binge-exposed) fetuses

**Table 6:** Associations between smoking, simultaneous smoking, and alcohol exposure and fetal growth (model 4, tobacco co-use model)

		Smoking—unadjusted^a^		Smoking + alcohol—unadjusted^b^			Smoking + alcohol—adjusted^b, c^	
	*n*	*ED Z*-score (95% CI)	*p-*value	*ED* Z-score (95% CI)	*p-*value	*n*	*ED Z*-score (95% CI)	*p-*value
**BPD**—**Z**								
20* weeks*	1150	**.0005 (.0002; .0008)**	** < .01**	.05 (− .07; .17)	.41	1142	.05 (− .07; .17)	.41
30 weeks		.0002 (− 1.6·10^−5^; .0005)	.07	− .07 (− .16; .03)	.16		− .05 (− .14; .04)	.29
36 weeks		.0001 (− .0002; .0004)	.52	− **.13 (**− **.25;** − **.02)**	**.02**		− .11 (− .22; .00)	.05
**HC**—**Z**								
20 weeks	1105	.0003 (− 4.8·10^−5^; .0006)	.02	− .02 (− .14; .10)	.72	1097	− .01 (− .12; .11)	.92
30 weeks		.0003 (− 1.4·10^−5^; .0005)	.04	− .07 (− .16; .01)	.10		− .06 (− .15; .02)	.16
36 weeks		.0002 (− .0001; .0005)	.21	− .10 (− .21; .01)	.06		− .09 (− .20; .01)	.09
**AC**—**Z**								
20 weeks	947	.0001 (− .0001; .0003)	.31	− .002 (− .10; .10)	.97	939	.00 (− .09; .10)	.95
30 weeks		.0002 (− 6.4·10^−6^; .0005)	.04	− .07 (− .14; − .01)	.07		− .05 (− .12; .02)	.16
36 weeks		.0003 (− 2.7·10^−5^; .0006)	.07	− **.11 (**− **.20;** − **.01)**	**.03**		− .09 (− .18; − 0.01)	.07
**FL**—**Z**								
20 weeks	1395	.0002 (− 9.5·10^−5^; .0005)	.19	− .11 (− .22; .01)	.06	1387	− .11 (− .22; .01)	.07
30 weeks		.0001 (− 9.8·10^−5^; .0004)	.24	− **.15 (**− **.24;** − **.06)**	** < .01**		− **.13 (**− **.22;** − **.04)**	** < .01**
36 weeks		.0001 (− 0.0002; .0004)	.43	− **.12 (**− **.28;** − **.07)**	** < .01**		− **.14 (**− **.25;** − **.04)**	** < .01**
**EFW**—**Z**								
20 weeks	847	.0002 (− 4.7·10^−5^; .0005)	.10	− .02 (− .13; .09)	.70	839	− .01 (− .12; .10)	.89
30* weeks*		.0003 (− 1.7·10^−5^; .0006)	.10	− .08 (− .16; .01)	.07		− .06 (− .14; .03)	.18
36 weeks		.0003 (− 5.6·10^−5^; .0006)	.10	− **.11 (**− **.21;** − **.003)**	**.04**		− .08 (− .19; .02)	.11

Estimated differences *(ED)* in *Z*-score were calculated for preset situations at GA 20, 30, and 36 weeks, comparing and exposed and non-exposed fetus

Abbreviations: AC, (fetal) abdominal circumference; *BPD*, (fetal) biparietal diameter; *CI*, confidence interval; *EFW*, estimated fetal weight; *FL*, (fetal) femur length; *HC*, (fetal) head circumference

^a^Alcohol exposure = 0; smoking = 10 cigs/day

^b^Alcohol exposure = 1 glass/day; smoking = 10 cigs/day

^c^Adjusted model settings for comparison; maternal age = 25 years, income = 910 SA Rand, anxiety score = 31, parity = 1, education = 10 years, fetal sex = “male”, other drug use = “yes”, MUAC = 277 mm

*n* is depicted per outcome, based on availability of growth measures, alcohol exposure, and gestational age

### Prenatal alcohol exposure, preterm birth, and hypertensive disorder

PAE was not associated with preterm birth (OR; 1.01 (0.99; 1.03), *p* = 0.29) or hypertensive disorders (OR; 1.00 (1.00; 1.00), *p* = 0.39).

## Discussion

### Principal findings

In this study, we showed a negative association of PAE with fetal growth. PAE, investigated as accumulative exposure over the course of pregnancy, was associated with reduced growth of the femur, abdomen, and lower birth weight. Also, trimester-specific PAE during the periconception period and second trimester was associated with reduced abdominal growth and a lower estimated fetal weight and birth weight. Third trimester PAE was associated with reduced femur growth and, unexpectedly, with an increased birth weight. Moreover, binge drinking was not additionally associated with fetal growth. Lastly, co-use of tobacco with alcohol was associated with a reduced femur growth.

### Results in the context of what is known

In line with our study, a shorter femur was found in the same cohort by Odendaal et al., measured at 34–38 weeks, and in a different cohort by Kfir et al. [18, 25]. In contrast to our study, Odendaal et al. investigated PAE in relation to fetal growth using categorized exposure variables. Some studies found no differences in AC and FL growth in alcohol exposed fetuses [16]. Differences in sample size (*n* < 100) and PAE prevalence (up to 37% in other studies vs. 62% in current study) could explain these disagreements [16, 18]. Also, the prevalences between other studies and this study are different. The average amount of maternal alcohol consumption in other studies, including different study populations was < 1 drink per week, compared to > 1 drink per week in our study population. Moreover, 27% of women in this study report binge drinking, compared to 0.08–0.4% binge drinking women in other studies [13, 16]. As hypothesized in the current study, most results show a negative direction, although not all statistically significant. Unexpectedly, we found no significant association between first trimester PAE and fetal growth, whereas periconceptional and second trimester alcohol exposure were negatively associated with fetal growth. A possible explanation lies in epigenetic changes in gametes due to periconceptional alcohol exposure influencing embryonic growth and placentation. As such, these epigenetic changes could influence fetal growth which is measurable later in pregnancy, as observed in this study at 30 and 36 weeks of gestation. Probably, the periconception period is more vulnerable regarding fetal growth, compared to first trimester in which possibly organ formation deficits could occur. Moreover, associations between second trimester exposure and fetal growth could be explained by the increased fetal growth potential, since it accelerates as gestational age progresses [43]. In line with previous research, accumulative and second trimester exposure was associated with lower birth weight [13]. Unexpectedly, we found an increase in birth weight after PAE during third trimester, which was also found in preterm newborns in another analysis in the same population [41]. Contrasting, another study in a bigger Safe Passage sub-cohort found smaller birth weight *Z*-scores in alcohol exposed fetuses, although Brink et al. investigated PAE as categorical variable, causing loss of information and possible misinterpretation of actual effect sizes [24]. Moreover, in our study, most women consumed alcohol during periconception period and first trimester, after which the amount of self-reported alcohol consumption decreases. In addition, of women drinking during third trimester, only one woman reported to consume more than 1 standard drink per day during the third trimester, indicating insufficient power to interpret this outcome with certainty.

In our study, no additional association between binge drinking and fetal growth was observed. Risky drinking behavior of binge drinkers, resulting in increased alcohol intake and fetal exposure could explain this null-finding [44]. Since our study population contains a high percentage of binge drinkers (27%), associations are probably embedded in the association between accumulative PAE and fetal growth. Tobacco exposure was high in our study population (up to 73%), compared to other countries. The major explanation could be found in differences in cultural practices, resulting in differences in lifestyle and attitude towards addictive substance use in general and also during pregnancy [45]. For example, the “Dop system,” known as a way of payment used in the past as partial compensation for labor, in the form of wine, might have introduced heavy alcohol consumption. Tobacco and alcohol are mostly co-used, explaining the high percentage of tobacco-users in this study population [31]. Simultaneous tobacco and alcohol exposure was associated with reduced femur growth, similar to previous research [32]. Previous research also found a reduced AC and BPD in third trimester in tobacco-exposed fetuses, which was not shown in our study [32]. However, these results should be interpreted with caution, since after correction for confounding factors, the effect sizes remaining in the FL measures are exactly similar to those in alcohol consuming women, as shown in Table 2 (model 1, the accumulative model). It could therefore be that the possible association found between simultaneous smoking and alcohol exposure with femur growth merely is a consequence of alcohol exposure.

In the current study, around 50% of pregnant women have depressive symptoms. This percentage is high compared to other studies, summarized in a systematic review reporting 7–13% of pregnant women having depressive symptoms [46]. Depression rates were found to be correlated with socio-economic status [47]. Women in our study population face high levels of poverty and unemployment (up to 67%), which could be an explanation for the high prevalence of depression.

### Possible mechanisms

Mechanisms explaining the relation between PAE and fetal growth are thought to be multifaceted and complex; however, exact molecular targets are unknown. First, it is hypothesized that growth restriction due to PAE arises from induced placental pathology. Alcohol reduces cellular proliferation, affects normal development of the placenta, and potentially results in reduced placental weight and underdeveloped blood vessels, impairing nutrient exchange between mother and fetus [48]. Second, genetic factors might influence growth deficiencies due to PAE. Studies investigating FASD report 9–14% of children with FASD have chromosomal deletions or duplications, partly explaining FASD features, including growth restriction [49]. Third, involvement of epigenetic reprogramming and environmental factors as nutrition have been proposed mechanisms influencing fetal growth [49]. Finally, PAE increases oxidative stress. Since in vivo treatment with antioxidants might reduce growth restriction, this is thought to be of influence, although the exact mechanism is not clarified yet [50].

### Clinical implications

Although our results demonstrate that the periconception period and second trimester are the most sensitive periods of exposure, we must be cautious in making interpretations about timing of alcohol consumption. Despite being at risk, most women report to quit or decrease alcohol consumption during pregnancy (Table 1), which could explain null-finding of third trimester PAE and fetal growth. Since this is a high-risk population regarding alcohol consumption, it is difficult to generalize these results to other populations. Worldwide, protocols about alcohol consumption in pregnancy are consistent in their advice about abstaining from alcohol. In practice, one in nineteen women consumes alcohol during pregnancy, of which 90% did not report this to their obstetric care giver [51]. Therefore, we think these results from a high-exposure study sample are important for populations with low-exposure populations as well. Thus, healthcare providers should actively discuss alcohol consumption, in a non-judging manner to comfort patients. Identifying risk factors (e.g., unplanned pregnancy, smoking and alcohol habits before pregnancy) might help health-care providers to effectively apply interventions including contingency management, cognitive behavioral therapy, and motivational interviewing [52, 53]. Moreover, it is important that healthcare providers educate patients about PAE, provide support, and promote abstinence, preferably already during the preconception phase [54].

### Research implications

FASD is a group of conditions indicating PAE alters brain development, which is mostly investigated using HC and BPD [10]. Our study found no association between PAE and HC or BPD growth, while others objectified a smaller HC in alcohol exposed fetuses [55]. To increase insight in detailed brain development of alcohol exposed fetuses, it is important to perform repeated ultrasound examinations during pregnancy, preferably with advanced techniques such as three-dimensional neurosonography [56, 57]. Additionally, different structures including the cerebellum, corpus callosum, thalami, and cortical folding processes should be examined [58–60].

### Strengths and limitations

The study strengths are the unique study population, the large sample, the high prevalence percentage of alcohol use during all three trimesters, and the heterogeneity in alcohol levels (i.e., low, moderate, high and binge drinking). Another strength is the assessment of alcohol exposure during pregnancy using the TLFB-method, resulting in alcohol consumption data with the best possible detail. The use of calendar worksheets and visual aids were used to help participants remember as much as possible about their alcohol consumption during a reference period of 30 days, minimizing recall bias as much as possible. Although this is retrospective data, using the TLFB-method resulted in detailed and complete data, compared to other studies. Also, the longitudinal study design measuring growth in different trimesters allows us to examine how PAE is associated with fetal growth rather than birth weight only.

Several limitations should be discussed. First is the stigmatization of substance abuse in general and during pregnancy being present in South Africa; this could lead to underreporting the actual intake [61, 62]. Despite the TLFB-method being the best possible method for investigation of alcohol consumption before or during pregnancy, objective measures to quantify alcohol exposure from biomarkers would be of added value to minimize underreporting of alcohol consumption. For example, in meconium, fatty acid ethyl esters (FAEE) and ethyl glucuronide (EtG) can be measured as marker for PAE. In a small substudy in the same study population, the latter was shown to have a significant dose-concentration relationship with self-reported drinks per drinking day [63]. Meconium starts to form between 12 and 18 weeks of gestation, providing long term information about PAE [64]. Another promising biomarker is phosphatidylethanol (PEth), recently studied in maternal blood and detects very low levels of alcohol consumption over the past 2–3 weeks [51]. Second, pregnancy dating in our study was mostly performed after 14 weeks of gestation, which is known to be less reliable. Accurate estimation of GA is essential to determine if fetal growth is appropriate and might result in unreliable growth measurements if not fulfilled. However, women were longitudinally assessed by trained sonographers using state-of-the-art ultrasound equipment, resulting in the most accurate growth measurements possible for this population. Additionally, the last series of ultrasound examinations (T3) were performed after 34 weeks of gestation (19% even after 36 weeks). The reliability of ultrasound examinations after 36 weeks of gestation is debatable; however, a recent cohort study showed that fetal ultrasound examinations at 36 weeks were more accurate in predicting birth weight compared to ultrasounds performed at 32 weeks [65]. Finally, although we aimed to correct for confounding factors, it might still be present due to nature of observational studies, and causal relationships cannot be studied.

## Conclusions

Our findings suggest that PAE is associated with reduced fetal growth, reflected by a smaller AC, FL, and EFW measured at 30 and 36 weeks of gestation and lower birth weight. Our results imply that the periconception period and second trimester might be the most sensitive periods regarding the negative impact of periconceptional and prenatal alcohol exposure on fetal growth. This study, especially with trimester specific analyses, provides an important insight in the relation between PAE and fetal growth, detectable before birth, and emphasizes the importance of alcohol abstention during pregnancy. Furthermore, this report should activate health care providers and policy makers to actively discuss PAE at every visit, educate patients, provide support, promote abstinence, or apply intervention strategies if necessary.

### Supplementary Information

**Additional file 1: Figure S1.** Flowchart of the study population.

**Additional file 2:** **Table S1.** Characteristics of included and excluded women in our study population. **Table S2.** Details of multiple imputation modelling.

**Additional file 3: Figure S2.** Distribution of alcohol consumption in study population. (Distribution of alcohol consumption in the alcohol exposed study group, depicted per exposure period).

**Additional file 4: Figure S3.** Fetal growth Z-score differences between fetuses exposed to alcohol during second trimester and non-exposed fetuses.

## Data Availability

The datasets used and/or analyzed during the current study are available from the corresponding author on reasonable request.

## Abbreviations

AC: Abdominal circumference
BMI: Body mass index
BPD: Biparietal diameter
CRL: Crown-rump-length
ED: Estimated difference
EFW: Estimated fetal weight
EPDS: Edinburgh postnatal depression scale
EtG: Ethyl glucuronide
FASD: Fetal alcohol spectrum disorders
FAEE: Fatty acid ethyl esters
FL: Femur length
GA: Gestational age
HC: Head circumference
MUAC: Mid-upper arm circumference
PAE: Prenatal alcohol exposure
PBC: Peak blood concentration
PEth: Phosphatidylethanol
TLFB: Timeline Followback method
