# Supplementary material for: Alcohol exposure before and during pregnancy is associated with reduced fetal growth: the Safe Passage Study
Source: BMC Med. 2023 Aug 23;21:318. doi: 10.1186/s12916-023-03020-4 (PMC10463675; doi:10.1186/s12916-023-03020-4)
Supplement: Supplementary file 1 — Additional file 1: Figure S1. Flowchart of the study population. [file 12916_2023_3020_MOESM1_ESM.docx]

**Additional file 1**

**Figure S1**. Flowchart of the study population

Women in substudy protocol in South Africa

n= 1928

Women with ultrasound data (growth and/or dopplers)

n= 1861

No ultrasound data

n= 67

Twin pregnancies n= 24

Termination of pregnancy n= 3

Stillbirth n= 25

Miscarriage n= 2

Missing growth data n= 24

Congenital anomaly n= 4

Multiple enrollments in study n= 81

**Total n= 163**

Study population

Singleton pregnancies

n= 1698
