# Supplementary material for: Alcohol exposure before and during pregnancy is associated with reduced fetal growth: the Safe Passage Study
Source: BMC Med. 2023 Aug 23;21:318. doi: 10.1186/s12916-023-03020-4 (PMC10463675; doi:10.1186/s12916-023-03020-4)
Supplement: Supplementary file 2 — Additional file 2: Table S1. Characteristics of included and excluded women in our study population. Table S2. Details of multiple imputation modelling. [file 12916_2023_3020_MOESM2_ESM.docx]

**Additional file 2**

|  | Included  (n= 1698) | Excluded  (n= 163) | *p*-value |
| --- | --- | --- | --- |
| *Maternal characteristics* | |  | |
| Age (years) | **24.8 ± 0.14** | **26.2 ±0.46** | **<.01** |
| Nullipara | **724 (42.6%)** | **28 (17.2%)** | **<.01** |
| BMI (kg/m^2^) | 25.3 ± 0.14 | 26.1 ± 0.48 | .09 |
| MUAC | 276.7 ± 1.15 | 282.3 ± 0.67 | .15 |
|  |  |  | |
| *Exposure to substances* |  |  | |
| Mean alcohol intake  periconception period (grams/day) | 2.30 ± 0.16 | 2.29 ± 0.67 | .98 |
| Mean alcohol intake 1^st^ trimester (grams/day) | 0.91 ± 0.07 | 0.93 ± 0.25 | .94 |
| Mean alcohol intake 2^nd^ trimester (grams/day) | 0.58 ± 0.04 | 0.80 ± 0.34 | .21 |
| Mean alcohol intake 3^rd^ trimester (grams/day) | 0.27 ± 0.02 | 0.38 ± 0.24 | .27 |
| Tobacco use during pregnancy (% users) | 1120 (66%) | 96 (58.9%) | .10 |
| Other drugs^a^ | 195 (11.5%) | 24 (14.7%) | .25 |
|  |  |  | |
| *Socio-economic characteristics* | |  | |
| Ethnicity^b^  Black  Cape colored (mixed ancestry) | 4 (0.2%)  1694 (99.8%) | 0 (0%)  163 (100%) | -  - |
| Income (monthly, South African Rand) | 927.7 ± 16.8 | 902.5 ± 46.3 | .60 |
| Employment (% employed) | 558 (32.9%) | 46 (28.2%) | .11 |
| Education (years) | **10.1 ± 0.04** | **9.8 ± 0.16** | **.01** |
|  |  |  | |
| *Prenatal psychopathology* | |  | |
| Anxiety scores | 30.9 ± 0.25 | 31.5 ± 0.84 | .47 |
| Depression (% above cut-off) | 854 (50.3%) | 68 (41.7%) | .07 |
|  |  |  | |
| *Pregnancy outcomes* | |  | |
| Gestational diabetes | 14 (0.8%) | 4 (2.5%) | .18 |
| Hypertensive disorder in pregnancy | 197 (11.6%) | 26 (15.6%) | .13 |
| Birth weight (grams) | **3012.3 ± 13.8** | **2468.4 ± 73.4** | **<.01** |
| Fetal sex (% female) | 872 (51.4%) | 87 (53.4%) | .68 |
| Preterm birth (% preterm) | **204 (12%)** | **61 (37.4%)** | **<.01** |
| GA at birth | **38.9 ± 0.05** | **36.1 ± 0.39** | **<.01** |
| Abbreviations: BMI, Body mass index; GA, Gestational age; MUAC, mid- upper arm circumference.  Note: Continuous data are presented as means ± standard errors; categorical variables are presented in numbers (%). Comparisons between non-exposed and exposed were performed using a Student’s t-test for normal distributed continuous variables, Mann-Whitney U test for non-normal distributed continuous variables and χ^2^ tests for categorical variables.  ^a^ Other drugs = marijuana or methamphetamine.  ^b^ Ethnicity was not compared due to very small subgroups. | | | |

**Table S1.** Characteristics of included and excluded women.

**Table S2.** Details of multiple imputation modelling.

| **Software used:** R (version 4.1.0; R Core Team (2021). R: A language and environment for statistical computing. R Foundation for Statistical Computing, Vienna, Austria - mice package (using 25 iterations) |
| --- |
| **Number of imputed datasets created:** 25 |
| **Variables included in the imputation procedure:**  Gestational age at different ultrasound examinations; alcohol exposure during different exposure periods in study; alcohol exposure overall; binge drinking; maternal smoking behavior during pregnancy; other drug use; maternal age; maternal BMI; maternal weight; maternal length; mid-upper arm circumference; maternal head circumference; gravidity; parity; total years of education; monthly income; anxiety symptoms during pregnancy; depressive symptoms during pregnancy; gestational diabetes in current pregnancy; hypertensive disorders in current pregnancy. |
