# Supplementary figures and images for: Alcohol exposure before and during pregnancy is associated with reduced fetal growth: the Safe Passage Study
Source: BMC Med. 2023 Aug 23;21:318. doi: 10.1186/s12916-023-03020-4 (PMC10463675; doi:10.1186/s12916-023-03020-4)

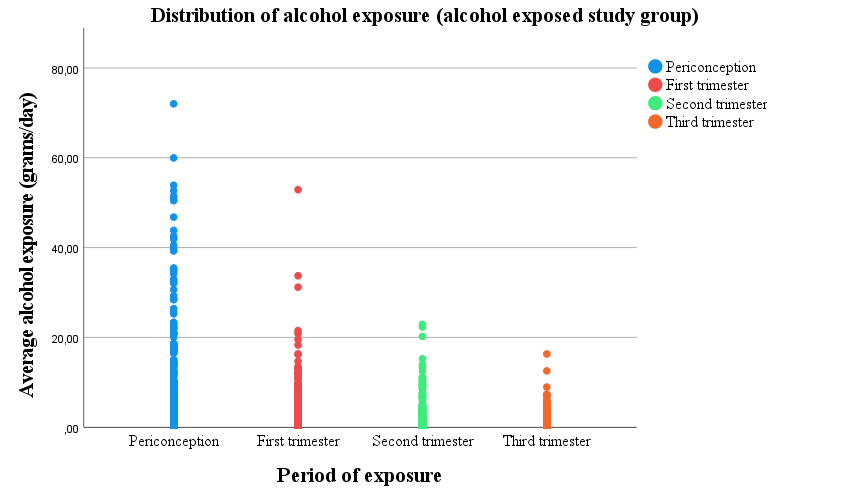

Supplement: Supplementary file 3 — Additional file 3: Figure S2. Distribution of alcohol consumption in study population. (Distribution of alcohol consumption in the alcohol exposed study group, depicted per exposure period). [file 12916_2023_3020_MOESM3_ESM.png]

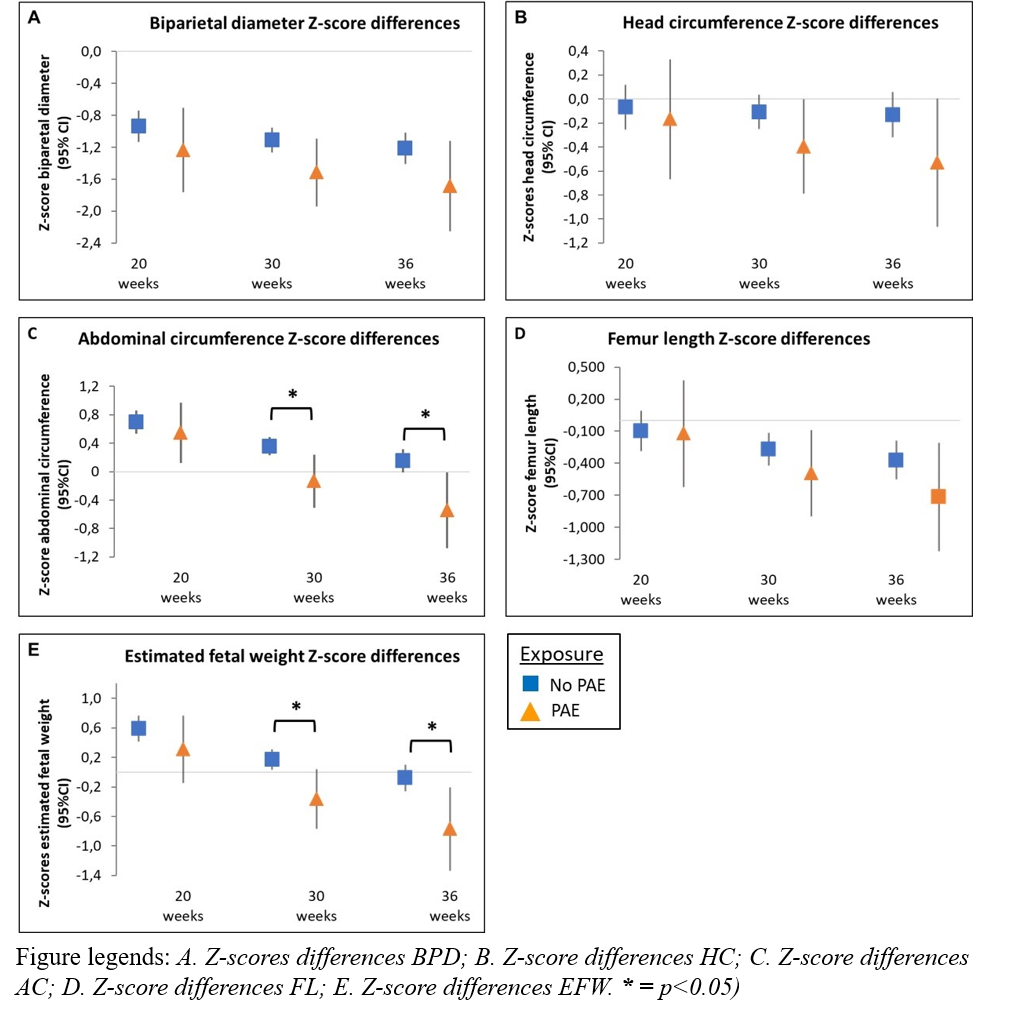

Supplement: Supplementary file 4 — Additional file 4: Figure S3. Fetal growth Z-score differences between fetuses exposed to alcohol during second trimester and non-exposed fetuses. [file 12916_2023_3020_MOESM4_ESM.png]
